# Sensitive Detection of Dengue Virus Type 2 E-Proteins Signals Using Self-Assembled Monolayers/Reduced Graphene Oxide-PAMAM Dendrimer Thin Film-SPR Optical Sensor

**DOI:** 10.1038/s41598-020-59388-3

**Published:** 2020-02-11

**Authors:** Nur Alia Sheh Omar, Yap Wing Fen, Jaafar Abdullah, Yasmin Mustapha Kamil, Wan Mohd Ebtisyam Mustaqim Mohd Daniyal, Amir Reza Sadrolhosseini, Mohd Adzir Mahdi

**Affiliations:** 1 Institute of Advanced Technology, Universiti Putra Malaysia, 43400 UPM Serdang, Selangor Malaysia; 2 Faculty of Science, Universiti Putra Malaysia, 43400 UPM Serdang, Selangor Malaysia; 3 inLAZER Dynamics Sdn Bhd, InnoHub Unit, Putra Science Park, Universiti Putra Malaysia, 43400 UPM Serdang, Selangor Malaysia; 4 Wireless and Photonics Network Research Centre, Faculty of Engineering, Universiti Putra Malaysia, 43400 UPM Serdang, Selangor Malaysia

**Keywords:** Optics and photonics, Physics

## Abstract

In this work, sensitive detection of dengue virus type 2 E-proteins (DENV-2 E-proteins) was performed in the range of 0.08 pM to 0.5 pM. The successful DENV detection at very low concentration is a matter of concern for targeting the early detection after the onset of dengue symptoms. Here, we developed a SPR sensor based on self-assembled monolayer/reduced graphene oxide-polyamidoamine dendrimer (SAM/NH_2_rGO/PAMAM) thin film to detect DENV-2 E-proteins. Surface characterizations involving X-ray diffraction (XRD) and Fourier-transform infrared spectroscopy (FTIR) confirms the incorporation of NH_2_rGO-PAMAM nanoparticles in the prepared sensor films. The specificity, sensitivity, binding affinity, and selectivity of the SPR sensor were then evaluated. Results indicated that the variation of the sensing layer due to different spin speed, time incubation, and concentration provided a better interaction between the analyte and sensing layer. The linear dependence of the SPR sensor showed good linearity (R^2^ = 0.92) with the lowest detection of 0.08 pM DENV-2 E-proteins. By using the Langmuir model, the equilibrium association constant was obtained at very high value of 6.6844 TM^−1^ (R^2^ = 0.99). High selectivity of the SPR sensor towards DENV-2 E-proteins was achieved in the presence of other competitors.

## Introduction

Dengue virus (DENV) is the most common arthropod-borne viral disease that poses a serious global problem. According to World Health Organization (WHO), the dengue virus is the leading cause of death of 22 000, annually. As of today, the need for hospitalization and medical treatment are constantly dense due to the fact that 390 million people in the world are still infected with DENV^1^. Its four distinct serotypes (DENV-1. DENV-2, DENV-3, and DENV-4) are capable of causing a range of clinical symptoms ranging from mild fevers to the severe dengue haemorrhagic fever (DHF) and be potentially life-threatening^2–10^. Despite its large burden to human health, no effective vaccine and antiviral therapy are available for the virus^11,12^. Early treatment for DENV is only by maintaining the body fluid of the patient, as it is critical in fighting the severe symptoms of DENV^13,14^. Hence, an early, rapid, and accurate diagnosis at the onset of infection is the demand of the day in the most epidemic settings.

Present discoveries in dengue diagnostics that can help in the early diagnosis are targeting the host-virus itself. DENV consists of a single-stranded positive-sense RNA virus that encodes 10 different types of proteins. Seven of them are NS1, NS2A, NS2B, NS3, NS4A, NS4B, and NS5, leaving the remaining three structural proteins of capsid, pre-membrane, and envelope (E) protein. Among these proteins, envelope glycoprotein, which also known as DENV E-proteins, has been proposed to be a therapeutic target for early detection at the DENV infection^15–22^. It is cleared that DENV E-proteins can mount sufficient immune response rapidly by producing detectable antibodies in patients for diagnosis.

Various detection techniques such as commercial NS1 kits, enzyme-linked immunosorbent assay (ELISA), and polymerase chain reaction (PCR), have employed as clinical dengue diagnostics^10,23–28^. Although these methods have made a significant breakthrough in targeting NS1 proteins, they have several obstacles. For example, commercial NS1 kits are restricted to the qualitative measurement of DENV, with the detection limit of 0.1 nM^29^, while for ELISA and PCR tests, their detection settings are slow, time-consuming, and also require the well-trained operators to handle the measurements^30–33^. In the past few decades, intensive efforts have been made towards the development of material-based biosensor for DENV detection^34–39^. The benefits offered by these advanced technologies including electrical sensors, fluorescent sensors, and optical fiber sensors are fast, sensitive and able to detect the DENV quantitatively with the detection limits of 0.019 pM^40^, 1 nM^41^, and 1 pM^42^, respectively. It then has attracted many researchers to lowering the detection limit of DENV to enable early-stage dengue diagnosis. However, increasing sensitivity and reducing the detection limit is still a priority today, hence, surface plasmon resonance (SPR) sensor would be a great choice for that. SPR has inherent advantages of being high-throughput, sensitive, label-free, economical, easy-to-use, and real-time monitoring^43–48^. In this regard, a great deal of work has been done in the exploitation of SPR biosensor for DENV detection^49–52^. Among recent studies include the detection of E-proteins using cadmium sulphide quantum dots-polyamidoamine dendrimer (CdSQDs-PAMAM) modified SPR gold film that achieved a detection limit of 0.1 pM and sensitivity of 5.0270 °/nM, showing a weak linear correlation of 0.18^53^. Therefore, there is a dire need for improved linear regression using graphene-SPR-based materials to achieve the best possible SPR sensitivity.

It is inevitable that the derivatives materials of graphene oxide including graphite oxide and reduced graphene oxide are the trending topic of recent research studies owing to their excellent properties. Graphite oxide is an oxidized form of chemically modified graphite, exhibiting some advantages of excellent corrosion resistance and low contact resistance. By exfoliation of graphite oxide, graphene oxide (GO) is produced with an abundance of oxygen-containing functional groups. Due to those properties, GO can be easily decorated with biomolecules as it has excellent dispersion in water, organic solvents, and different matrixes^54^. With respect to electrical conductivity, this electrically insulating graphene oxide can be reduced to produce reduced graphene oxide (rGO). The advantages of rGO have over GO as it can be stored longer without agglomeration, more stable in organic solvents, and inferior electrical properties^55–57^. Interestingly, some oxygen groups on the rGO surface may be benefit for chemical functionalization in the preparation of composite material. Along these lines, functionalized rGO with primary amine (−NH_2_) renders them to be hydrophilic and increase its interfacial binding to materials of interest, thus, making it more adaptable as a sensing platform for detection of dengue virus^58–60^. Likewise, combining a globular shaped of polyamidoamine (PAMAM) dendrimer into rGO provides great opportunities to enhance the sensitivity of detection. PAMAM dendrimers are believed to have the advantages in sensing applications due to their high binding site on the dendrimer and high efficiency of transporting bioactive agents^61–64^. This in return created more active sites for the attachment of DENV E-proteins.

In this work, a strategy was carried out by self-assembling the sensor surface with dithiobis (succinimidyl undecanoate) (DSU) for immobilization of NH_2_rGO-PAMAM nanocomposite. The SPR measurements such as specificity, sensitivity, binding affinity, selectivity of the proposed sensor would be discussed. To the best of our knowledge, this is the first report on the lowest detection of DENV-2 E-proteins at a concentration of 0.08 pM within 8 minutes using DSU/NH_2_rGO-PAMAM thin film-based SPR optical sensor.

## Methodology

### Chemicals

Dithiobis (succinimidyl undecanoate) with a molecular weight of 628.84 g/mol was used as SAM layer, purchased from Dojindo Japan. Graphene oxide was purchased from Graphanea, Spain. PAMAM dendrimer (ethylenediamine core, generation 4.0 solution in methanol), Ethylenediamine (EDA), N-hydroxysuccinimide (NHS), bovine serum albumin (BSA) was purchased from Sigma Aldrich, Germany. N-Ethyl-N-(3-(dimethylaminopropyl) carbodiimide (EDC) was bought from Fluka, Switzerland. The purified, recombinant dengue virus type 2 E-proteins (DENV-2 E-proteins) with a concentration of 2.94 mg/ml and its dengue virus type 2 E-proteins monoclonal antibodies (IgM) with a concentration of 1 mg/ml were ordered from Meridian Life Science. Phosphate buffered saline (PBS), which is used in the dilution process, was prepared by compounding the solution of Na_2_HPO_4_ and NaH_2_PO_4_. All chemicals were of reagents or higher grade, and deionized water was used throughout the experiments.

### Fabrication of Au/DSU/NH_2_rGO-PAMAM/IgM sensor film

Glass substrates (Menzel glass, 2.4 cm × 2.4 cm) were cleaned using acetone. Subsequently, a thin gold layer was sputtered onto the glass substrate using SC7640 Sputter Coater (I = 20 mA). The gold-coated substrates were rinsed with water and followed by rinsing in ethanol and dried in a nitrogen flow. The substrates were then immersed in DMSO solutions of 2 mM DSU for 24 h. Afterward, the substrates were thoroughly rinsed with acetone and PBS solution (pH 7.4), and the Au/DSU surface was ready to use in the next procedure. Approximately 0.50 ml of the NH_2_rGO-PAMAM composite solution was first dropped onto the substrate surface. After 30 min, the substrate was spun using Spin Coating System, P-6708D. After spinning, the mixture of EDC/NHS was incubated onto the substrate surface for another 30 min, accompanied by the further spinning process. Following that, the substrate surface was further activated and covered by 50 µl of antibodies specific to DENV-2 E-proteins (0.01 µM in PBS, pH 7.4) with a surface area of approximately 57.6 cm^2^ to form an amide bond. The duration of incubation was 30 min, followed by spinning at a speed of 5000 rpm for 20 s.

### Materials characterization

XRD patterns of the synthesized NH_2_rGO, and NH_2_rGO-PAMAM composite were recorded on X-ray Diffractometer (Philips X’Pert X-ray) with the Cu Kα radiation. The surface functionalization of the proposed sensor film was then confirmed using Fourier transform infrared (FTIR) spectrometer (VERTEX 70) in the wavenumber range of 4000–400 cm^−1^.

### Surface plasmon resonance (SPR) sensing

**Figure 1:**
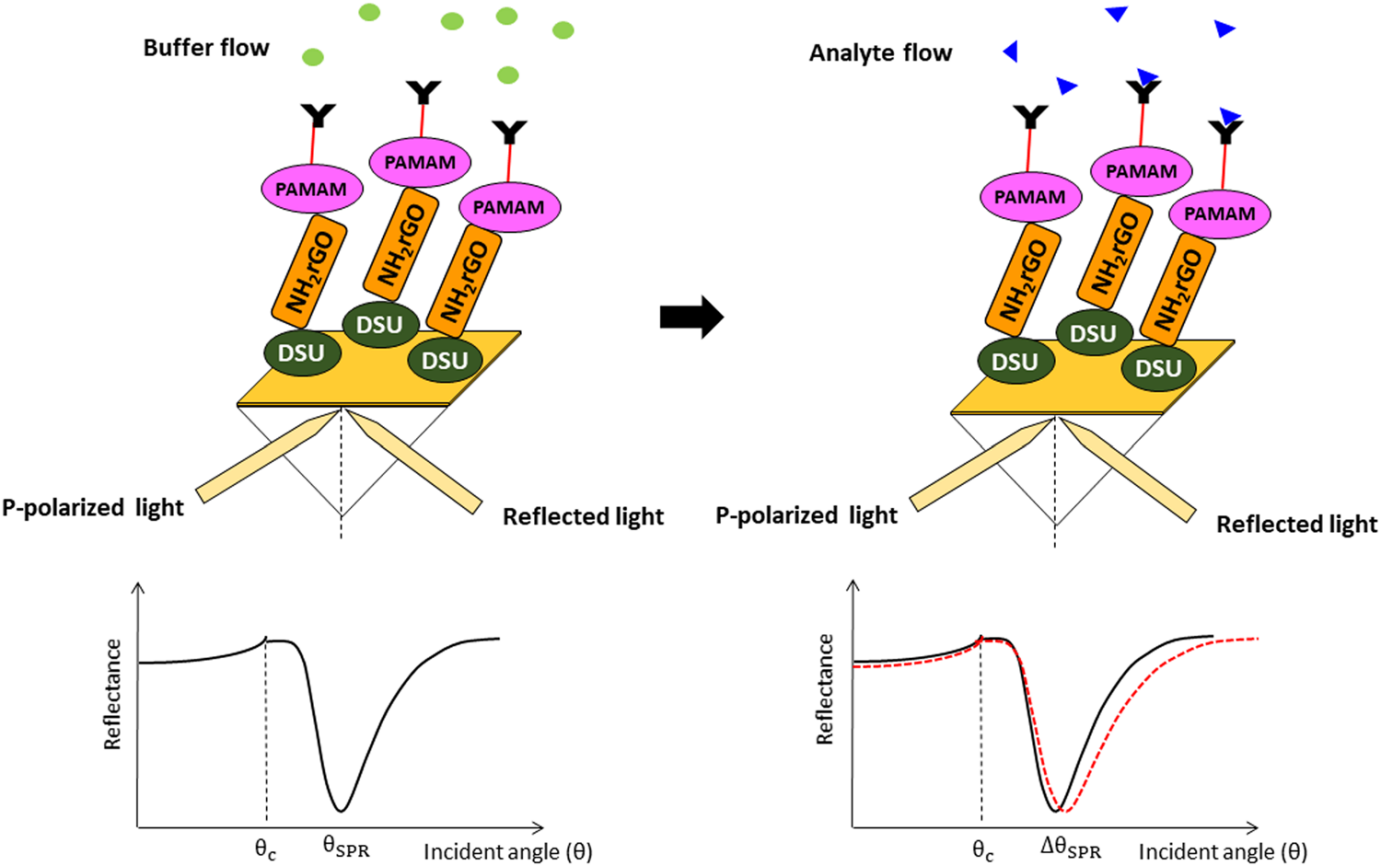
Schematic illustration of the SPR signal before and after the analyte flow.

SPR measurements were conducted based on Kretschmann configuration^65–68^ by evaporating Au/DSU/NH_2_rGO-PAMAM/IgM sensor film onto the prism surface. The prism was placed on an optical stage driven by a stepper motor with a resolution of 0.001° (Newport MM 3000) to let the incident light from laser beam (632.8 nm, 5 mW) pass through the prism and hits a gold layer to generate the surface plasmon waves at the interface. At a specific angle of the incident light, the SPR response was induced when the evanescent wave is generated due to the change in the refractive index of the medium in close to the vicinity of a gold surface. The SPR response is the reflected light intensity, at which its angle at minimum intensity was recorded with time. A 100 µl flow cell was attached to the sensor film to be filled up by DENV-2 E-proteins solution for the detection system. All experiments were conducted at room temperature and replicated three times with a new set of sensor surface for each concentration of DENV-2 E-proteins (0.08 pM – 0.5 pM). Figure 1 is a schematic illustration representing the proposed Au/DSU/NH_2_rGO-PAMAM/IgM sensor with the introduction of DENV-2 E-proteins.

## Results and Discussion

### Materials characterization

**Figure 2:**
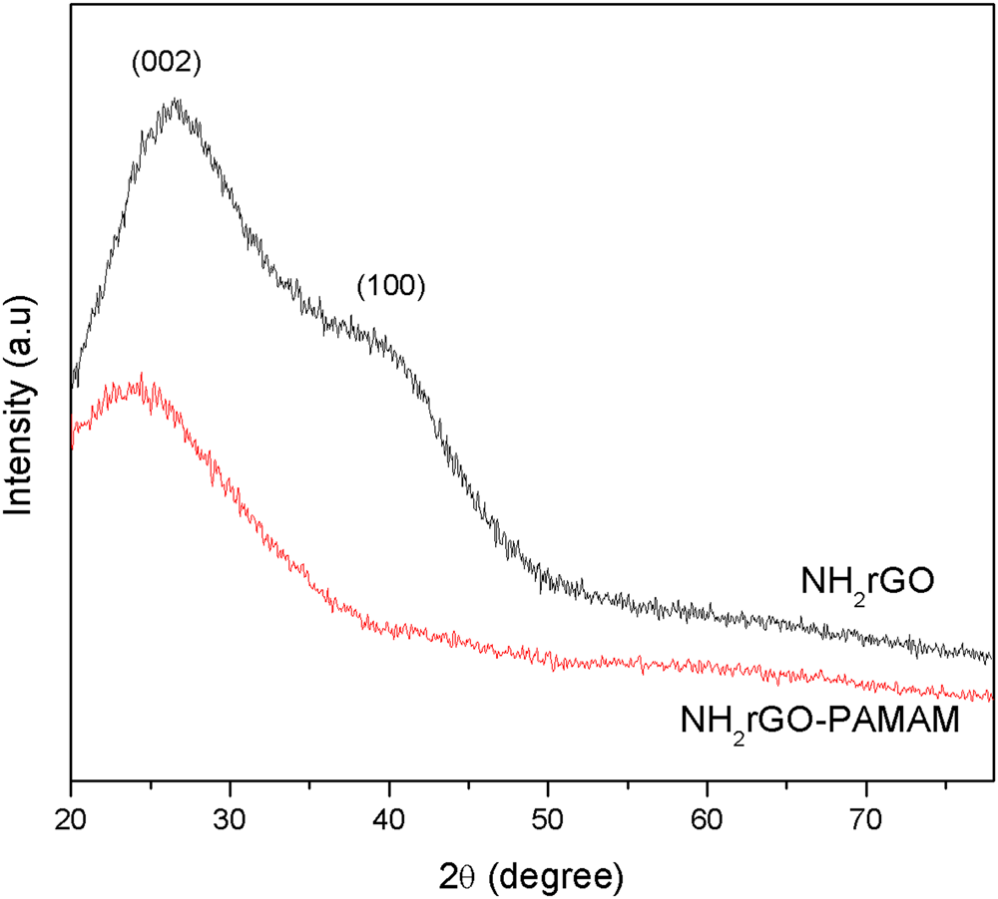
XRD pattern of the synthesized NH_2_rGO, and NH_2_rGO-PAMAM composite.

Figure 2 depicts the XRD patterns of the synthesized NH_2_rGO, and NH_2_rGO-PAMAM composite. The formation of the synthesized NH_2_rGO was confirmed by a broad diffraction peak at 2θ = 26.35 and a small hump at 2θ = 40.17°, which stand for the (002) and (100) crystal plane of NH_2_rGO^69–72^. After being composited with PAMAM, the amorphous nature of NH_2_rGO was restored and slightly shifted to the left. This shift can be associated to the changes in lattice parameters caused by the covalent binding between PAMAM and NH_2_rGO.

**Figure 3:**
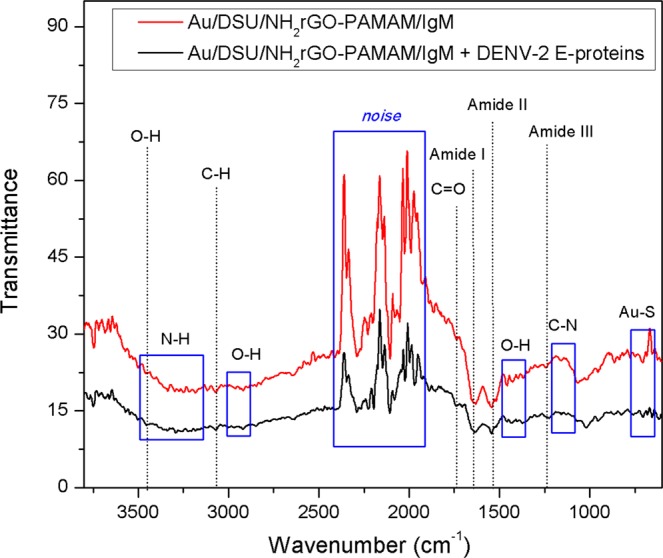
FTIR spectra of Au/DSU/NH_2_rGO-PAMAM/IgM sensor film before and after the introduction of DENV-2 E-proteins.

Characterization was continued using FTIR spectroscopy to examine the chemical structure of the Au/DSU/NH_2_rGO-PAMAM/IgM thin film before and after antigen conjugation, as shown in Fig. 3. From the FTIR spectra, the absorption band of Au-S at 650–720 cm^−1^ confirms the adsorption of a self-assembled monolayer on a gold film. The peaks at 1570 cm^−1^ and 1631 cm^−1^ is related to the N-H bending (amide II) and C-O stretching (amide I) vibration of PAMAM dendrimer, respectively^73^. Three peaks at 3448, 2912, and ~1400 cm^−1^ are observed that can be indicated to the stretching of adsorbed water molecules and structural O-H groups in graphene oxide. In the case of rGO with amine functionalized, the bandwidth at 3200–3400 cm^−1^ belonged to the N-H stretching, which overlapped with O-H bonds^74^. Another overlapped peak observed at 1550 cm^−1^ and a small peak at 1150 cm^−1^ were attributed to N-H bending and C-N stretching, respectively, hence confirms the amine functionalization of GO followed by reduction to amino-rGO^59^. After covalent bonding with the antibodies via EDC/NHS, a small peak of C=O stretching could be observed at 1716 cm^−1^, with regard to certifying the amide bonding^75,76^. This proved that the bioconjugation proceeded successfully. Furthermore, with the introduction of DENV-2 E-proteins into the sensor film, the reduction in the intensity of amide bands and O–H band can be observed quantitatively, indicate the structural changes of the sensor surface due to binding. This finding thus validates the immunoreaction between antibodies immobilized on the sensor surface with the DENV-2 E-proteins has taken place.

### Optimization of SPR sensing layers

**Figure 4:**
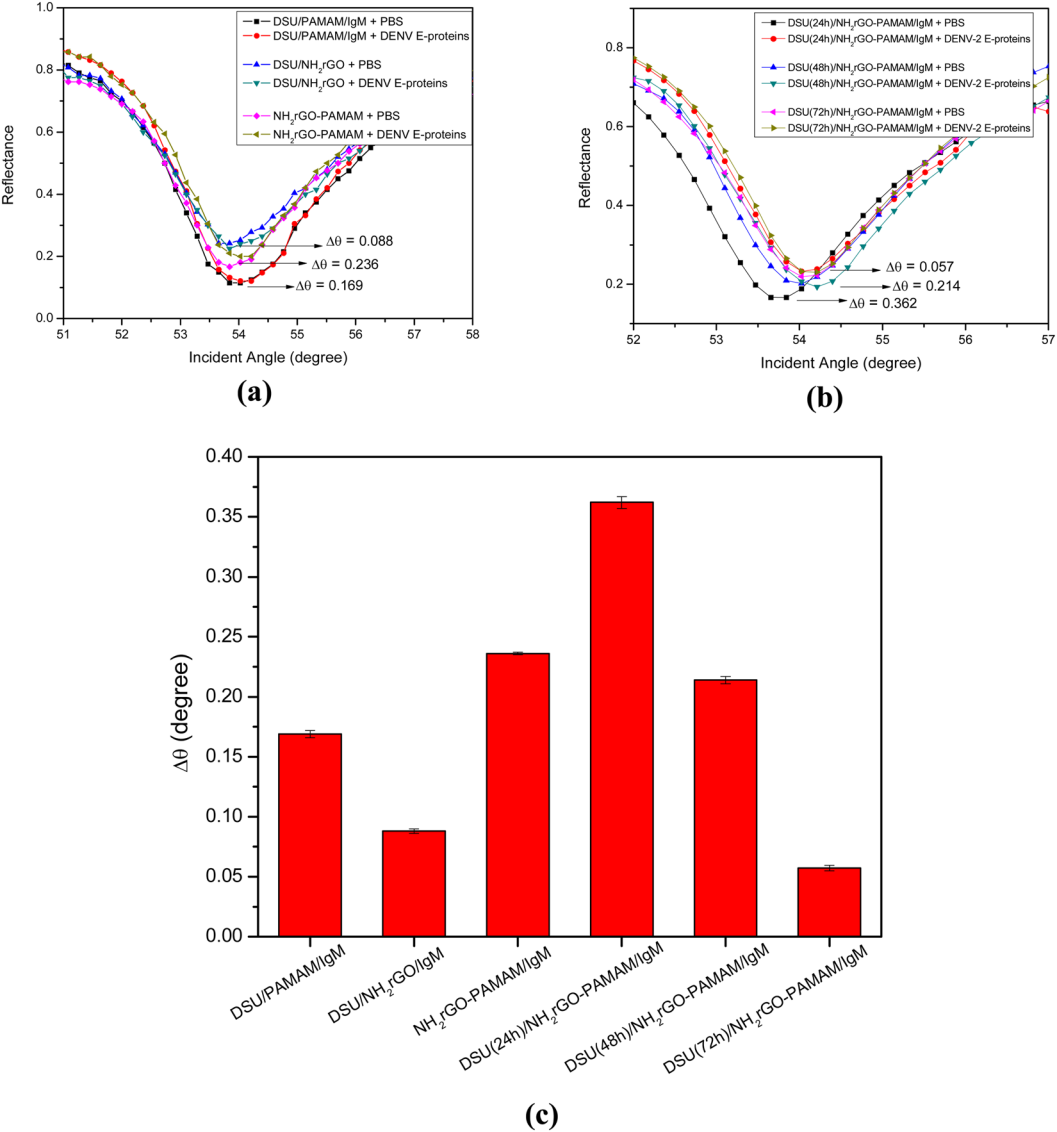
Detection of 100 pM of DENV-2 E-proteins on (**a**) different types of composite sensor layers and (**b**) different incubation times for self-assembly. (**c**) Results are expressed by a bar graph.

The excellent performance of SPR sensor lies in its sensor surface functionalization, which offers a significant binding towards DENV-2 E-proteins. Significant binding of antigens results in a change in the refractive index of the medium in close to the vicinity of a gold surface, which in turn is measured as a shift in the SPR angle^77,78^. To achieve the best SPR enhancement performance, we plotted the shift in resonance angle as a function of various fabricating material-coated gold films as shown in Fig. 4(a,b), i.e. DSU/PAMAM/IgM, DSU/NH_2_rGO/IgM, NH_2_rGO-PAMAM/IgM, DSU(24 h)/NH_2_rGO-PAMAM/IgM, DSU(48 h)/NH_2_rGO-PAMAM/IgM, and DSU(72 h)/NH_2_rGO-PAMAM/IgM. As shown in Fig. 4(c), the binding between DENV-2 E-proteins and sensing surface of DSU(24 h)/NH_2_rGO-PAMAM/IgM resulted in an obvious shift in the SPR resonance angle. Furthermore, when comparing to the last three sensing surfaces which differ in self-assembly time optimization, the assembly time of 24 h gives the highest shift in the resonance angle. This is because the longer incubation time than 24 led to the desorption of SAM structures. This indicates that DSU(24 h)/NH_2_rGO-PAMAM/IgM sensing layers can serve as the best dengue sensing medium in SPR technique, thus offering a significant enhancement in the penetration depth of evanescent waves.

### Effect of PAMAM concentrations

**Figure 5:**
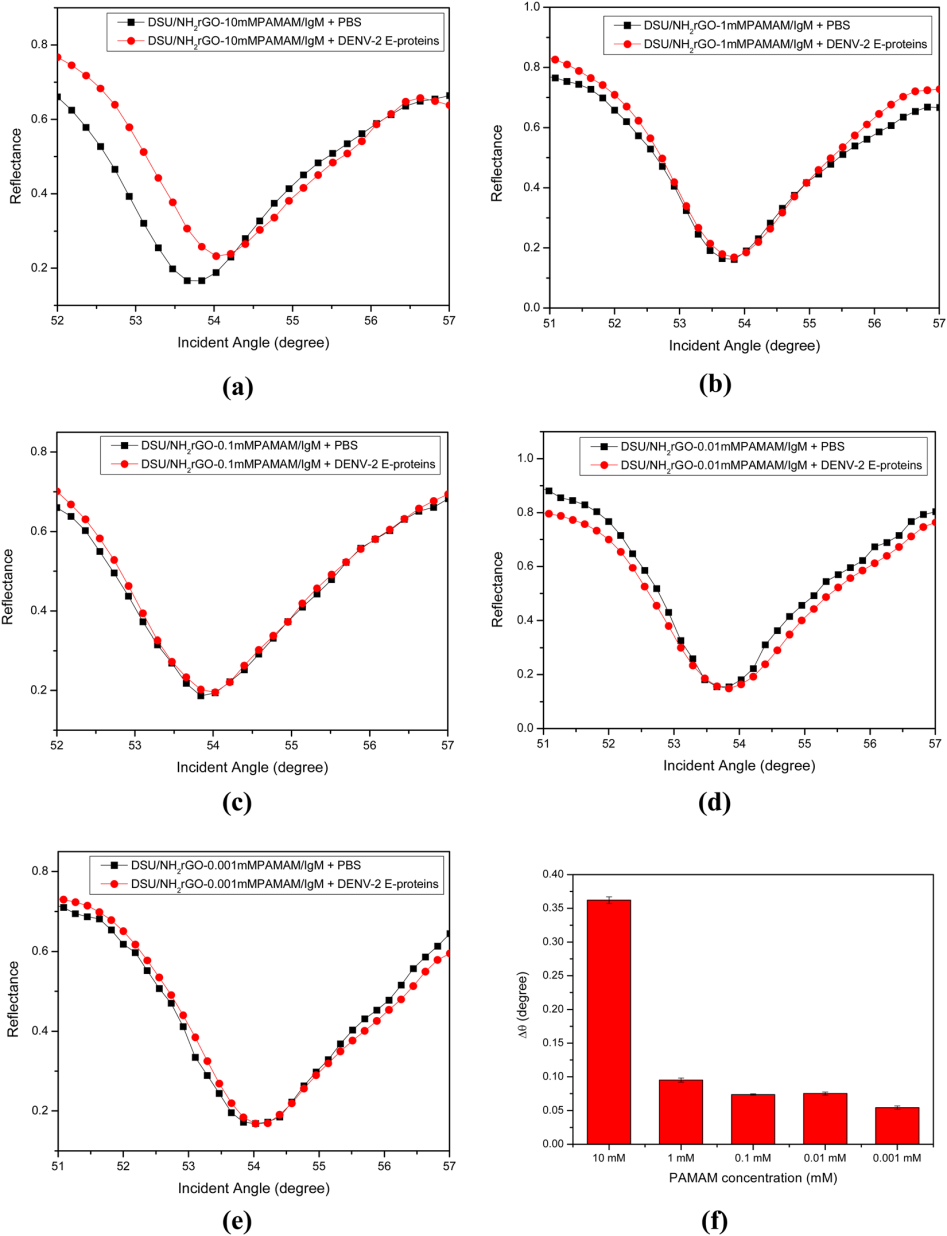
Detection of 100 pM of DENV-2 E-proteins on different concentration of PAMAM dendrimer: (**a**) 10 mM, (**b**) 1 mM, (**c**) 0.1 mM, (**d**) 0.01 mM, and (**e**) 0.001 mM. (**f**) Results are expressed by a bar graph.

In order to establish the optimal condition for the sensing layer, we varied the concentrations of PAMAM dendrimer (Fig. 5). From the SPR experiments, the shift in the resonance angle increased dramatically with PAMAM concentration of 10 mM. The higher value in Δθ obtained for 10 mM PAMAM dendrimer showed significant improvement in the detection of DENV-2 E-proteins. Perhaps, the effect of a higher concentration of PAMAM dendrimer can be attributed to the higher activity of dendrimer-encapsulated reduced graphene oxide. As a result, the presence of dense concentration and globular shape of dendrimer might have provided better interaction between antigen and sensing layer^79^. Therefore, 10 mM PAMAM dendrimer was established as optimal concentration, which is in agreement with those reported in the literature^80^.

### Effect of sputtering time and spin speed

**Figure 6:**
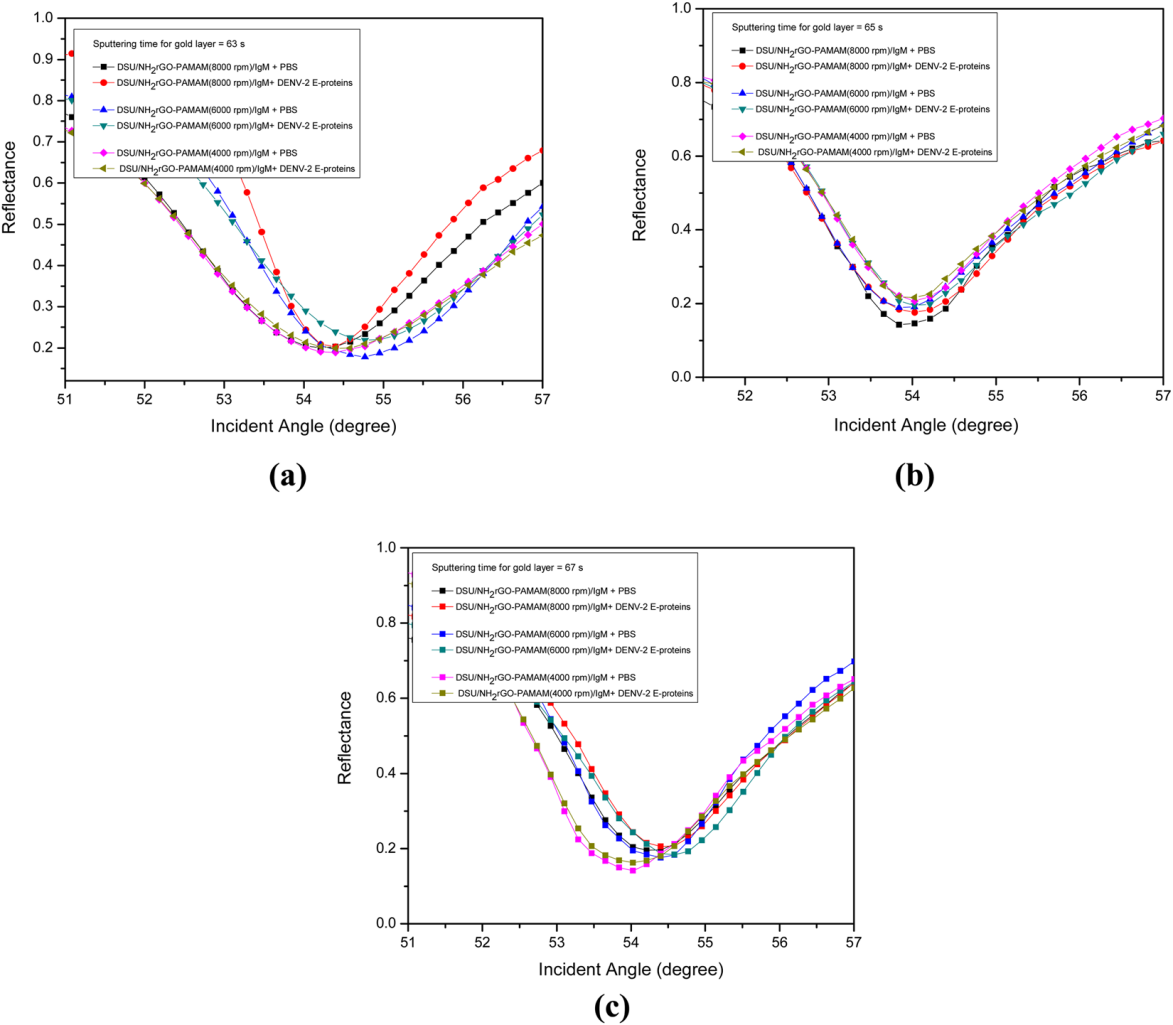
Detection of 100 pM of DENV-2 E-proteins on different sputtering time for a gold layer: (**a**) 63 s, (**b**) 65 s, and (**c**) 67 s.

**Figure 7:**
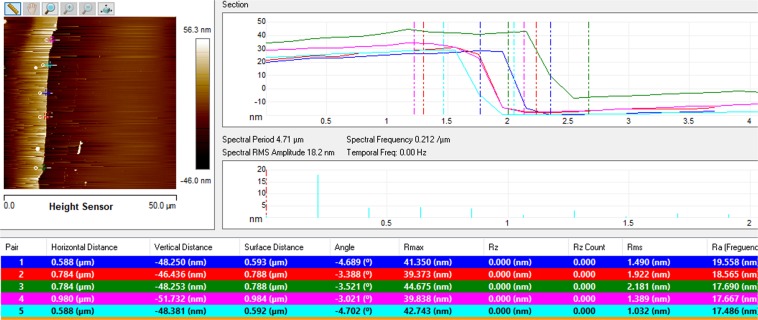
Thickness of the gold layer obtained from AFM analysis.

In order to achieve a relatively high shift in resonance angle and low width of the resonance curve, we optimized the sputtering time for gold layer and spin speed of NH_2_rGO-PAMAM layer (Fig. 6). The important of width (i.e., FWHM) lies in determining the resonance angle accurately^81,82^. From Table 1, it is noted that as the sputtering time for gold was fixed at 67 s with the increasing spin speed of deposition, the reflectance curve is redshifted and narrower. The increases in resonance angle indicate that the sensor layer tends to absorb the biomolecules as they become thinner. In addition, the interaction between evanescent field and sensing medium has resulted in the deeper penetration depth of the field in the biomolecular analyte layer. In this case, the propagation constant (wavevector) of surface plasmons will be enhanced due to smaller electron energy loss. Comparing with the sputtering time of 65 s and 63 s, the smallest FWHM was achieved at 63 s. The reason behind this is might be due to the less scattering near surface plasmon, thus has selective detection towards DENV-2 E-proteins. Regardless of the narrower FWHM at sputtering time of 63 s, the optimum sputtering time for the gold layer and spin speed of NH_2_rGO-PAMAM layer were 67 s and 8000 rpm, respectively. A significant increase in FWHM could be due to the intensified internal loss as resulted from the higher binding of target antigen^83,84^. On the other hand, the average thickness of the gold layer at 67 s was found to be ~48 nm, confirmed by AFM analysis as shown in Fig. 7 (see vertical distance). It can also be mentioned the obtained thickness is nearer to the most typical configuration in the SPR system with a 50 nm gold film, which is responsive to the local gold-dielectric interface^85–87^.

**Table 1:** The optimized values of sputtering time for gold layer and spin speed of NH_2_rGO-PAMAM layer. The concentration of DENV-2 E-proteins was 100 pM.

Sputtering time for gold layer (s)	Spin speed of NH_2_rGO-PAMAM layer (rpm)	Δθ (degree)	FWHM (degree)
63	4000	0.1690	2.7723
	6000	0.0079	2.5412
	8000	0.1621	2.0081
65	4000	0.0554	2.2441
	6000	0.2268	2.2604
	8000	0.0894	2.3096
67	4000	0.0212	2.5352
	6000	0.2087	2.3460
	8000	0.2577	2.2325

### Comparison of SPR signals for DENV-2 E-proteins detection

**Figure 8:**
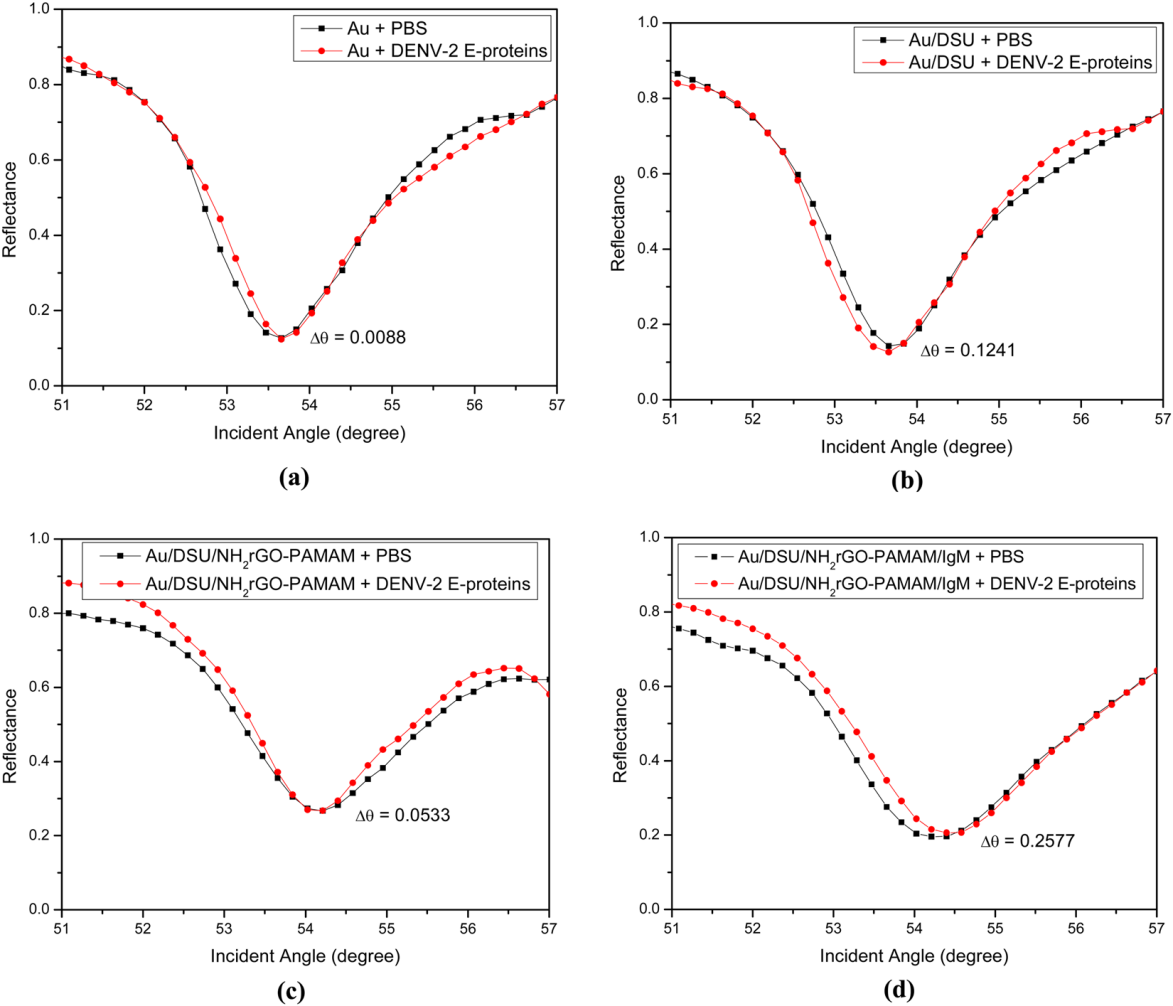
Comparison of four differently SPR signals on modified SPR sensor surfaces generated by introduction of 100 pM of DENV-2 E-proteins: (**a**) Au film, (**b**) DSU alone, (**c**) DSU/NH_2_rGO-PAMAM, and (**d**) DSU/NH_2_rGO-PAMAM/IgM.

Figure 8a–d display the variation of SPR signal on modified SPR sensor surfaces generated by the introduction of 100 pM DENV E-proteins. As observed in Fig. 8a, the first bare gold film shows the resonance angle shifts from 53.6554° to 53.6642° upon the introduction of target antigens. Further, the resonance angle was blue-shifted after the bare gold film was self-assembled with the amine-reactive sites of DSU (Fig. 8b). The remarkable blueshift of the resonance curve after injection of DENV solution can be explained by the dissociation of the target antigen towards the sensor surface. As the composite layer of NH_2_rGO-PAMAM was developed onto the sensor surface (Fig. 8c), a decreasing shift in resonance angle was observed. This leaves the sensor surface requires specific biomolecules for selective detection of DENV solution. When a solution containing antibodies specific to DENV E-proteins is immobilized on the sensor surface (Fig. 8d), the shift in resonance angle is greatly enhanced to 0.2577° which results in a significant change in SPR sensitivity. Therefore, complete development of sensor film, Au/DSU/NH_2_rGO-PAMAM/IgM is responsible for selective and sensitive detection of target antigens as they have a great change in SPR angle.

### SPR reflectivity with real-time measurement

**Figure 9:**
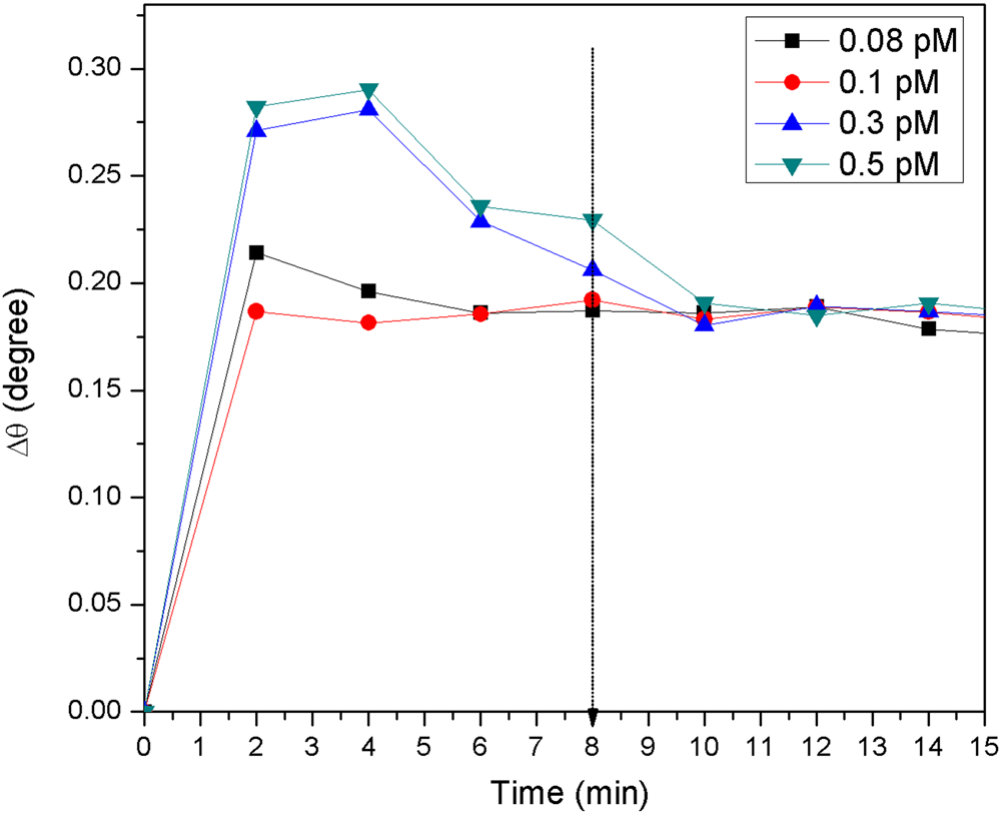
Real-time detection for different concentrations of DENV-2 E-proteins in contact with Au/DSU/NH_2_rGO-PAMAM/IgM sensor film.

Figure 9 shows the SPR real-time measurement of the proposed Au/DSU/NH_2_rGO-PAMAM/IgM sensor film for detection of dengue virus. To verify the viability of the sensor film, different concentrations of DENV-2 E-proteins in the range of 0.08–0.5 pM were injected accordingly into the cell. The proposed sensor had a complete response time at approximately 10 min for detection of higher DENV-2 E-proteins concentration, while 6–8 min for lowest concentration of DENV-2 E-proteins, 0.08 pM. It indicates that an increase in concentration caused an increase in real-time detection of DENV-2 E-proteins, which might be due to saturation of all binding sites. Due to that, all DENV-2 E-proteins concentrations were left for 8 min before the SPR response was taken.

**Figure 10:**
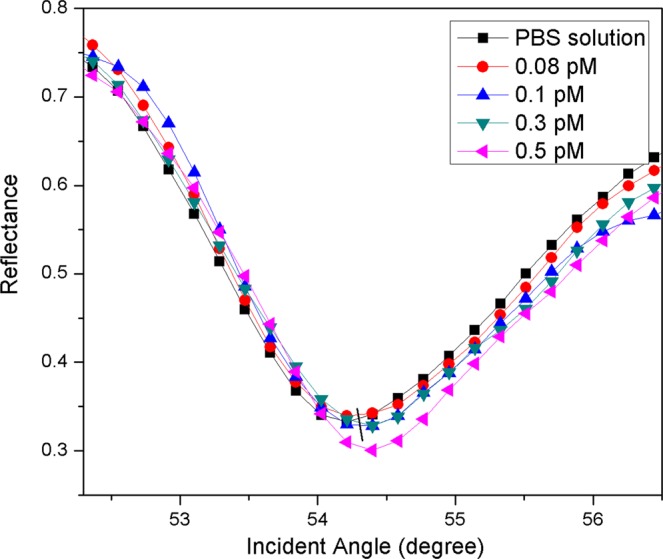
Experimental SPR curves when Au/DSU/NH_2_rGO-PAMAM/IgM sensor film was exposed to 0.08 pM-0.5 pM of DENV-2 E-proteins.

Figure 10 shows the SPR responses of the proposed sensor film for detection of DENV-2 E-proteins. The results showed that the resonance angle for PBS solution was 54.2138°. When the proposed sensor was exposed to the lowest concentration of DENV-2 E-proteins (0.08 pM), the resonance angle of the reflected light increased to 54.3052°. Subsequently, the resonance angles from SPR curves were found to be 54.3137°, 54.3925°, and 54.4004° with further addition of DENV-2 E-proteins concentrations of 0.1 pM, 0.3 pM, and 0.5 pM, respectively. To measure the number of antigens bound to sensor surfaces, the resonance angle shift (Δθ) was taken from the difference in resonance angle of antigen and resonance angle of the reference solution. It was found that the rise in Δθ of 0.0914°, 0.0999°, 0.1708°, and 0.1866° were obtained for detection of 0.08 pM, 0.1 pM, 0.3 pM, and 0.5 pM, of DENV-2 E-proteins, respectively. These Δθ can be attributed to the changes in the refractive index of the sensor surface which in turn changes the real part of the dielectric constant of the gold film caused by the binding of DENV-2 E-proteins. It was inferred that a change of the thickness of the sensing layer also brings out a slight angle shift of SPR as the evanescent wave possesses longer penetration depths^88,89^.

### Sensitivity and binding affinity of DSU/NH_2_rGO-PAMAM/IgM sensor film

**Figure 11:**
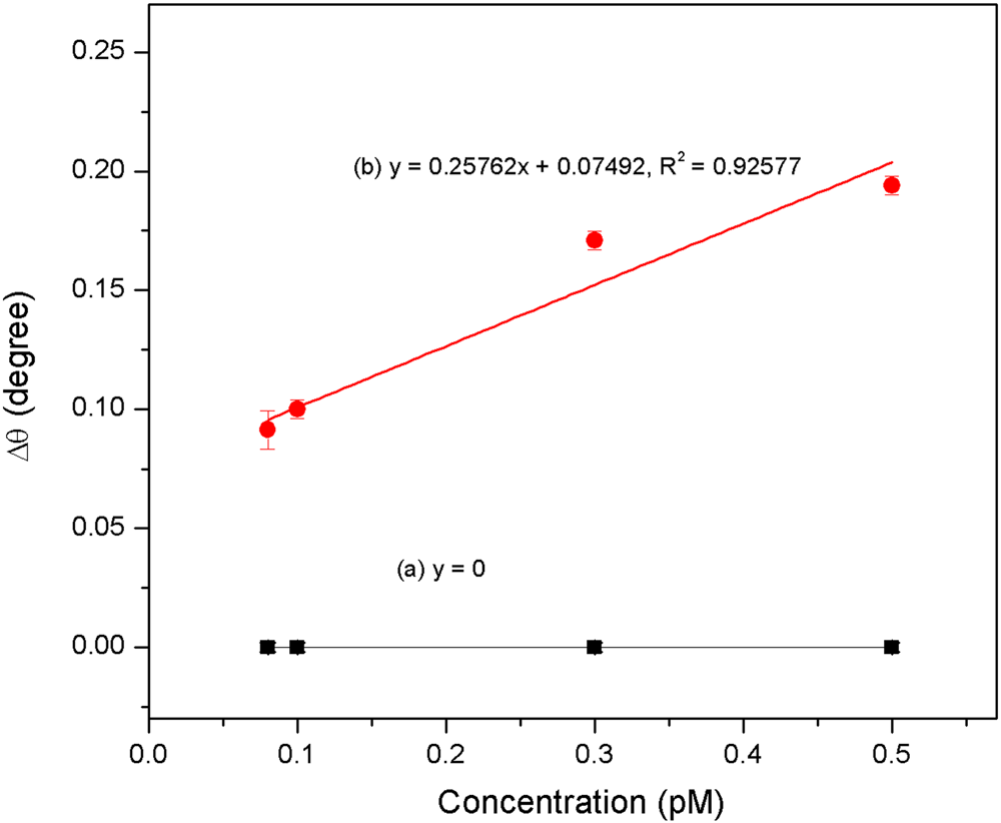
The dependence of the (**a**) Au/IgM and (**b**) Au/DSU/NH_2_rGO-PAMAM/IgM sensor response on the DENV-2 E-proteins concentrations ranging from 0.08 pM to 0.5 pM.

Prior to the sensitivity measurement of Au/DSU/NH_2_rGO-PAMAM/IgM sensor film, a control experiment was performed using Au/IgM. For this purpose, different concentrations of DENV-2 E-proteins ranging from 0.08–0.5 pM were injected onto Au/IgM surface. Table 2 depicts the obtained resonance angle shift in a triplicate manner. It was observed that there were no variations in the SPR resonance angle with average standard deviation value of ±0.0004. Further, the linear regression analysis for Au/DSU/NH_2_rGO-PAMAM/IgM sensor film was plotted as shown in Fig. 11, yielded y = 0.25762x + 0.07492; R^2^ = 0.92 with average standard deviation value of ±0.0044. From a comparison of the gradient for Au/IgM and Au/DSU/NH_2_rGO-PAMAM/IgM, the gradient of Au/DSU/NH_2_rGO-PAMAM/IgM reveals higher sensitivity than Au/IgM. The results indicated that DENV-2 E-proteins can be sensitively detected at the lowest concentration of 0.08 pM using Au/DSU/NH_2_rGO-PAMAM/IgM based SPR sensor.

**Table 2:** The shift of resonance angle for different concentrations of DENV E-proteins in contact with Au/IgM and Au/DSU/NH_2_rGO-PAMAM/IgM sensor film.

Sensor film	Sample (pM)	Shift of resonance angle, Δθ (°)			Sensitivity (°/pM)	Average standard deviation (±)
		Δθ_1_	Δθ_2_	Δθ_3_		
Au/IgM	0.08	0	0	0	0	0.0004
	0.1	0	0	0		
	0.3	0	0.0001	0.0020		
	0.5	0.0020	0.0015	0.0009		
Au/DSU/NH_2_rGO-PAMAM/IgM	0.08	0.0834	0.0914	0.0931	0.2576	0.0044
	0.1	0.0903	0.0999	0.1039		
	0.3	0.1668	0.1708	0.1738		
	0.5	0.1899	0.1866	0.1904

**Figure 12:**
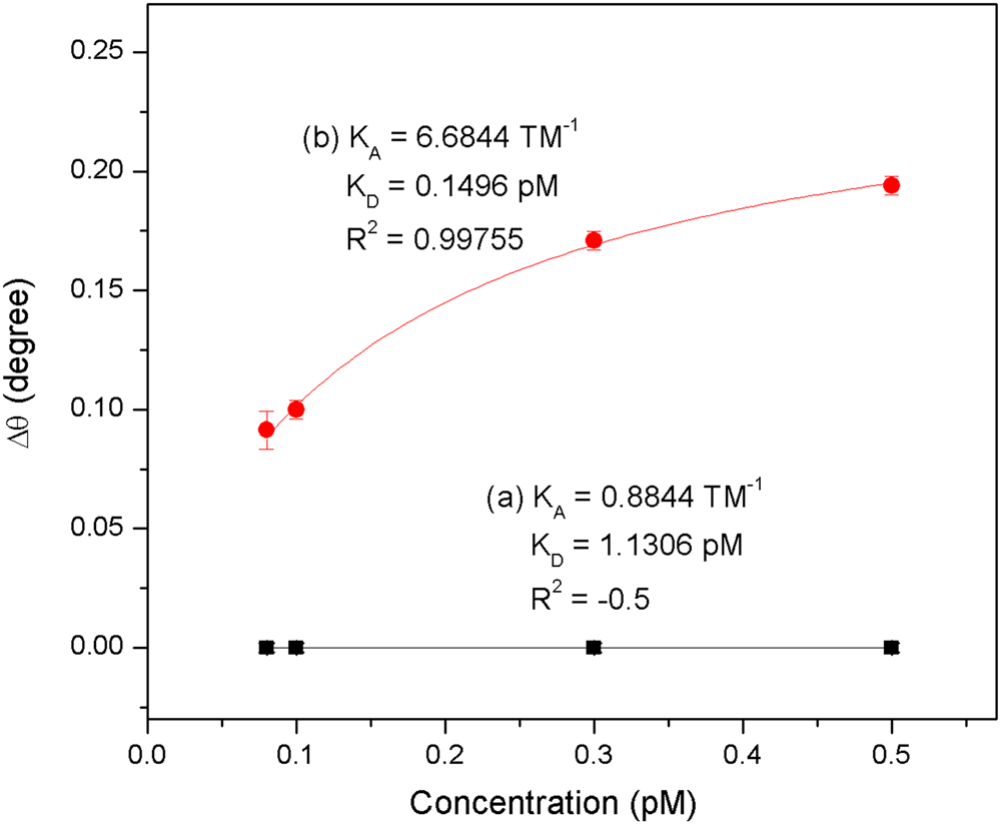
Binding affinity between (**a**) Au/IgM and (**b**) Au/DSU/NH_2_rGO-PAMAM/IgM sensor film and DENV-2 E-proteins concentrations ranging from 0.08 pM to 0.5 pM.

To extract the binding strength involved in analyte-ligand interactions, the data of SPR angle shifts (Δθ) and DENV-2 E-proteins concentrations were fitted using Langmuir isotherm model (Fig. 12). The equation of this model is represented by^90,91^

1 $$\Delta \,\theta =\frac{\Delta \,{\theta }_{{\rm{\max }}}C}{{K}_{D}+C}$$

where *Δθ*_*max*_ is the maximum SPR shift at the saturation, *C* is the concentration of DENV and *K*_*D*_ is the equilibrium dissociation constant. The K_D_ for the assessment of the DENV-2 E-proteins towards Au/IgM and Au/DSU/NH_2_rGO-PAMAM/IgM were then calculated and found to be 1.1306 pM; R^2^ = −0.5 and 0.1496 pM; R^2^ = 0.99, respectively. The obtained K_D_ values are found to be consistent with the standard K_D_ value for protein interaction (K_D_ < 10 nM)^92,93^. The smaller K_D_ value revealed that the Au/DSU/NH_2_rGO-PAMAM/IgM sensor film has higher affinity (K_A_) interaction towards DENV E-proteins compared with Au/IgM sensor film. The K_A_ values for Au/IgM and Au/DSU/NH_2_rGO-PAMAM/IgM sensor films were then calculated to be 0.8844 TM^−1^ and 6.6844 TM^−1^, respectively. The cause of these changes was significantly due to differences in ligand density and stability, which can potentially affect the evanescent field distribution at the gold interface, thus affect the quantitative detection of the target analyte. It thus validated that the integration of DSU/NH_2_rGO-PAMAM/IgM sensor layer into SPR gold film is important to improve the detection of DENV-2 E-proteins.

Table 3 presents the performance comparison of other reported SPR-based DENV sensor in terms of sensitivity (S), dissociation constant (K_D_), and detection limit. The proposed SPR sensor in this work shows excellent DENV detection performance including the highest sensitivity and binding affinity, lowest detection limit, and faster response times within 8 minutes. It is hypothesized that modification of the SPR-gold thin film would have an influence on the surface mass associated with a binding event and consequently cause a significant change in the SPR signal.

**Table 3:** Performance comparison of the previously reported using SPR-based DENV sensor.

SPR substrate	Surface modification	Target	Sensitivity, S and dissociation constant, K_D_	Limit of detection
Gold^49^	Carboxymethylated dextran/N-ethyl-N-(dimethylaminopropyl) carbodiimide-N-hydroxysuccinimide (EDC-NHS)	IgM	K_D_ = 5 nM	1 nM
Gold^29^	Carboxymethylated dextran/EDC-NHS	DENV-2 NS1	K_D_ = 12.3 nM	0.25 ng/mL
Gold^50^	16-mercaptohexadecanioc acid/EDC-NHS	IgM	—	12 pg/mm^2^
Gold^51^	16-mercaptohexadecanioc acid/EDC-NHS	NS1	—	5.73 pg/mm^2^
Gold^52^	Carboxymethylated dextran/EDC-NHS	IgM	S = 0.0132 pM/sec^−1^ K_D_ = 2.12^−12^ nM	2.125 pM
Gold (our work)	Dithiobis (succinimidyl undecanoate)/reduced graphene oxide-polyamidoamine dendrimer/EDC-NHS	DENV-2 E-proteins	S = 0.25762 °/pM K_D_ = 0.1496 pM	0.08 pM

### Selectivity study

**Figure 13:**
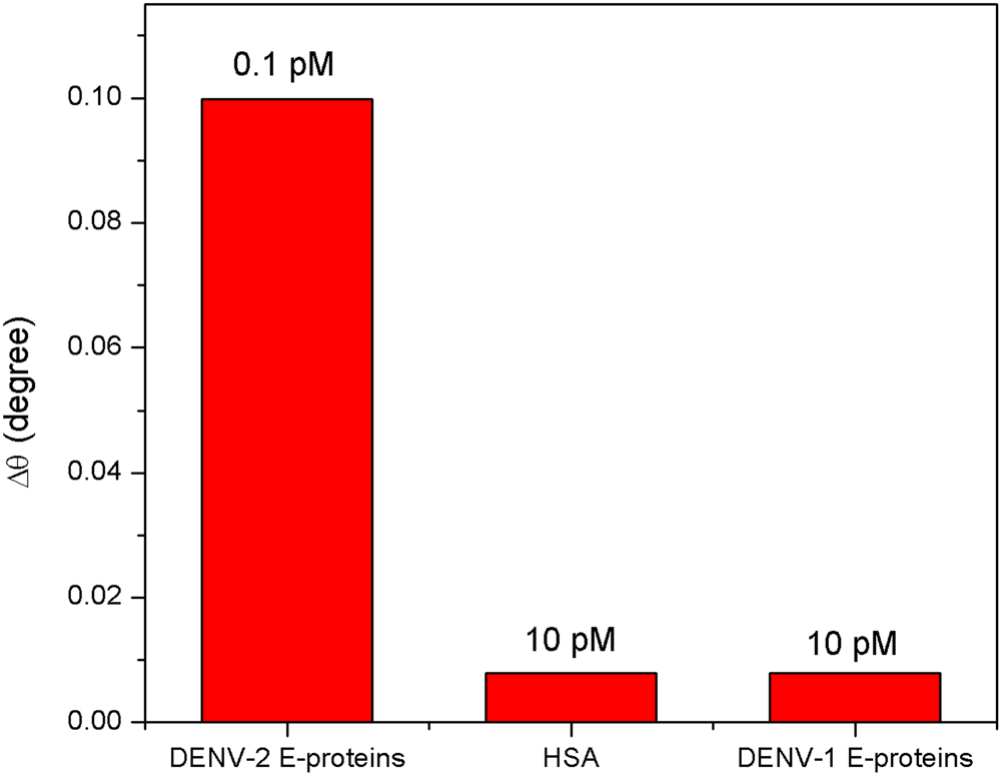
Selectivity test of Au/DSU/NH_2_rGO-PAMAM/IgM sensor with different target antigens.

The selectivity of the proposed SPR sensor film toward DENV-2 E-proteins was also investigated relative to other potentially competitive proteins such as HSA and DENV-1 E-proteins (Fig. 13). The tests revealed that the sensor response to 0.1 pM DENV-2 E-proteins was higher than of other proteins. One can conclude that the Au/DSU/NH_2_rGO-PAMAM/IgM-based SPR sensor film has good selectivity to DENV-2 E-proteins. This remarkable selectivity may be due to the high affinity between DENV-2 E-proteins and its specific antibodies immobilized on the sensor surface. It was later verified that the NH_2_rGO-PAMAM sensor layer is developed to increase the adsorption of antibodies to provide more active sites for attachment of DENV-2 E-proteins. In the case of HSA proteins and DENV-1 E-proteins, the results indicated a least SPR response due to the non-specific antibody binding. The least response from DENV-1 E-proteins likely reflects the successful interactions of similar genome, which shares 65% of single-stranded RNA genomes encoded by other DENV serotypes^94,95^. As for HSA proteins, high SPR response can be accounted as an excessive proteins in the blood with a molecular weight of 66.4 kDa when compared to 50 kDa DENV-1 E-proteins^96,97^.

**Figure 14:**
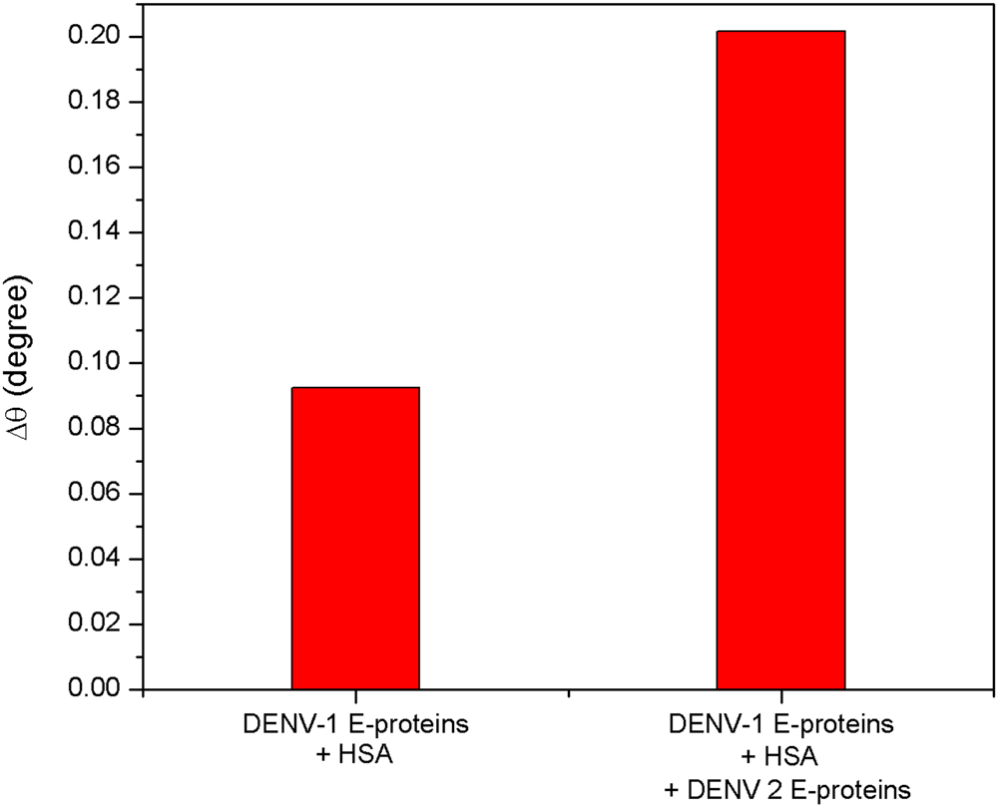
Selectivity test of Au/DSU/NH_2_rGO-PAMAM/IgM sensor in different mixture of analytes.

Figure 14 depicts the effect of diverse analyte solutions on the selective detection of DENV-2 E-proteins using SPR sensor. The concentrations of each analyte were fixed at 10 pM. The multiple solutions that having DENV-2 E-proteins showed larger SPR signal compared to other solutions containing no DENV-2 E-proteins. The results suggest that the interference by other analyte solutions does not affect the quantitative detection of DENV-2 E-proteins.

## Conclusions

In this work, a high sensitivity of SPR sensor was successfully developed for detection of DENV-2 E-proteins by self-assembling a gold surface with dithiobis (succinimidyl undecanoate) (DSU) for immobilization of NH_2_rGO-PAMAM nanocomposite. The results revealed that the developed SPR sensor could successfully detect the lowest concentration of DENV-2 E-proteins of 0.08 pM within 8 min with a sensitivity and binding affinity values of 0.2576° pM^−1^ and 6.6844 TM^−1^, respectively. The sensor also showed high selectivity towards DENV-2 E-proteins. Taken together, this Au/DSU/NH_2_rGO-PAMAM/IgM thin film-integrated SPR sensor can be a useful dengue diagnostic for the development of point-of-care devices in the future.
